# The Relationship Between Perceived Social Support and PTSD Symptoms After Exposure to Physical Assault: An 8 Years Longitudinal Study

**DOI:** 10.1177/0886260520970314

**Published:** 2020-11-03

**Authors:** Venke A. Johansen, Anne Marita Milde, Roy Miodini Nilsen, Kyrre Breivik, Dag Øystein Nordanger, Kjell Morten Stormark, Lars Weisæth

**Affiliations:** 1 Haukeland University Hospital, Bergen, Norway; 2 Western Norway University of Applied Sciences (HVL), Bergen, Norway; 3 NORCE Norwegian Research Centre AS, Bergen, Norway; 4 University of Bergen, Norway; 5 Oslo Metropolitan University, Oslo, Norway; 6 University of Oslo, Norway

**Keywords:** perceived social support, PTSD, physical assault, cross-lagged, longitudinal, nondomestic violence

## Abstract

Consequences of nondomestic violence are known to be multifaceted with high rates of emotional and psychological problems in addition to physical injuries, and victims report many trauma related symptoms. This study explore if perceived social support (PSS) (Social Provisions Scale [SPS]) and post-traumatic stress disorder (PTSD) symptoms (Impact of Event Scale [IES-22]) are interrelated among adult victims at four assessment points up until eight years after the exposure to physical assault; soon after the event (T1), three months after T1 (T2), one year after T1 (T3), and eight years after T1 (T4). One hundred and forty-three subjects participated at T1, 94 at T2, 73 at T3, and 47 accepted a follow-up at T4. At T1, 138 of 143 completed the questionnaires within 16 weeks after the incident. PTSD symptoms were highly correlated across time (*p* < .001); PSS were significantly correlated only between T1 and T2 (*p* < .001), T1 and T3 (*p* < .05), and between T2 and T3 (*p* < .05). Cross-lagged analyses showed an inverse relationship between prior PSS and later PTSD symptoms across all time points (*p*s < .05); not proved between prior PTSD symptoms and later PSS (*p*s > .1). PSS at T1 was an independent predictor of PSS one year and eight years after the incident. We conclude that higher perception of social support protects against the development of PTSD symptoms; diminished perception of social support increases the risk of developing PTSD symptoms. These findings suggest that PSS after experiencing a violent assault should be considered as an important factor in natural recovery in the long run, as well as essential alongside psychiatric treatment. Establishing psychosocial interventions for victims of physical violence in the acute phase may prevent prolonged trauma reactions.

## Introduction

It is well documented that consequences of interpersonal violence for crime victims represent a significant public health problem (Kilpatrick et al., 2003; >WHO, 2002). We have however sparse knowledge on the long-term consequences on mental health among adults exposed to physical assault by a perpetrator who is not an intimate partner, ex-partner, or close family. Acute distress, as well as prolonged post-traumatic stress disorder (PTSD) symptoms predicts poor mental health across lifespan (Olatunji et al., 2010; Sareen, 2014). Exposure to physical assault combined with actual physical injury and perceived fear of being more seriously injured or killed during the event, are major risk factors for developing PTSD (Kilpatrick & Acierno, 2003; Sareen, 2014). Most of the affected victims do recover within weeks or months, but 10%–40% develop persistent PTSD lasting for years or even for life (Sareen, 2014). Exposure to intentional interpersonal violence is more likely to conduct PTSD symptoms than accidents or disasters (Holbrook et al., 2001; Stein et al., 1997). Moreover, intentional or assaultive injury, among others, has shown to be a risk factor for the onset of PTSD (Sareen, 2014).

Several studies have highlighted the relationship between social support (SS) and PTSD symptoms after trauma-exposure (Brewin et al., 2000; Ozer et al., 2003; Peleg & Shalev, 2006; Vogt et al., 2017). Most literature include perceived positive SS and empathy from significant others as protective factors, while blaming and social exclusion are presented as risk factors (Maercker & Horn, 2013; Vogt et al., 2017). Two important metastudies concluded respectively that lack of SS and support of poor quality were among the most potent peri- and post-trauma risk factors for the development of PTSD (Brewin et al., 2000; Ozer et al., 2003).

Defining SS in the aftermath of potential traumatic events (PTE) may be of challenge. The phenomenon of SS may be understood both in terms of its multifaceted, interactive nature, as well as how it is perceived (Pruitt & Zoellner, 2008). Though some studies focus upon actual versus perceived social support (PSS), the latter seem more frequently reported (Guay et al., 2011). The value of SS seem to be more dependent of the recipients’ perception of the interaction, than of the intention from the other person trying to provide support (Pruitt & Zoellner, 2008). It can be health protective if the recipient perceives others as reliable during stressful experiences. Positive support can be understood as relationships offering information assistance, material or emotional help, and a sense of cohesion that are perceived as loving or caring by the victim (Hobfoll & Stephens, 1990; Hollifield et al., 2016). The protective role of PSS from close others is well documented (Brewin et al., 2000; Vogt et al., 2017). Negative SS, on the other hand, includes blame, disbelief, taking control of the victim’s choice and withdrawal from the beneficiary. Absence of SS is defined as a lack of any reaction from others (Pruitt & Zoellner, 2008).

It seems important to get a broader view of the individual interactions in different contexts (Vogt et al., 2017). Maercker and Horn (2013) have constructed a sociointerpersonal model that organize the individual’s involvement within different levels of social contexts. The model includes three levels of contextual and interpersonal processes that arise after exposure to traumatic events. The first level, “Individual” (social-effective responses), consists of intrapersonal features or impairments. The second level “Close social relationships” occur during interactions between the victim and those who share a close relationship that in adulthood is typically represented by a romantic partner, close family members, and friends. The third level “Distant social contexts” (culture and society) is based on a shared culture, religion, or society, where the interactions are related to a specific group. The model reflects transactional or reciprocal relationships between the different levels.

The interplay between SS and PTSD symptoms needs to be scrutinized, as SS includes many perspectives with a variety of relations to PTSD symptoms (Guay et al., 2011). In explaining the association between SS and PTSD symptoms, two main sets of theories are predominant in the literature field. One is the social causation theory that explains SS in terms of its antecedent effect on PTSD symptoms: negative/poor SS can lead to impaired mental health and increased psychopathology, while positive support protects against development of PTSD symptoms (Joseph et al., 1997; Mossakowski, 2014). The second way of annotation includes social selection theories (also known as social deterioration and social erosion theory) embracing that SS is affected by poor mental health (Wagner et al., 2016; Woodward et al., 2018).

According to theories of social causation, favorable SS may have protective effects, while adverse social conditions such as low SS, small networks, social isolation or low socioeconomic status, may increase the risk of PTSD (Mossakowski, 2014; Woodward et al., 2018). Emotional support, validation, and involvement with significant others in a noncritical context are often found to be protective and helpful (Ozer et al., 2003; Scarpa et al., 2006). Interactions with others, who intentionally give negative and unsupportive responses, for instance by blaming or excluding the victim, are found to have negative impacts. When relatives who are expected to be supportive, have opposite reactions to what is expected by the victim, for example by blaming or taking control, it can undermine the victims’ self-confidence (Scarpa et al., 2006; Ullman & Siegel, 1996). In sum, receiving SS may have a positive or caring effect, but while experienced as criticism, consequences may be induced or maintained distress both immediately after an adverse event or in the long run (Andrews et al., 2003; Guay et al., 2011).

The social selection theories propose that PTSD symptoms such as insecurity, mistrust, avoidance and social isolation, can unfold rejection and less SS from others (Mossakowski, 2014). In addition, PTSD symptoms can break down social relationships through an increased number of negative social interactions as well as through erosion of social resources in close relationships across time (Freedman et al., 2015; Wagner et al., 2016; Woodward et al., 2018). This perspective thus addresses that developing PTSD symptoms may increase the risk of experiencing negative dyadic communication, lesser networks, reduced support, in addition to cause an inability for the victim to improve poor SS (Freedman et al., 2015; Woodward et al., 2018). Further, caregiver burden and secondary traumatization can disturb such communication thus underline the complexity of social interaction (Lambert et al., 2012).

Social causality and social selection theories has previously been understood as two competing explanations (Kaniasty & Norris, 2008). However, gradually these theories have been presented as being more complementary thus contributing to the discussion of a potent association between SS and PTSD symptoms and its implications for general health (Shallcross et al., 2016). Evidence from longitudinal studies support a bidirectional relationship between SS and PTSD symptoms in adults, involving processes from both theories (Kaniasty & Norris, 2008; Shallcross et al., 2016; Woodward et al., 2018).

The majority of studies among adult crime- or community-victims exposed to physical assault have been cross-sectional (Kaniasty & Norris, 2008; Vogt et al., 2017), and among those, only a few report findings on both SS and PTSD symptoms (Jaycox et al., 2003; Scarpa et al., 2006; Yap & Devilly, 2004). Longitudinal studies including cross-lagged analysis on the relationship between PSS and PTSD symptoms in adult civilians seem scarce (Kaniasty & Norris, 2008; Woodward et al., 2018). Cross-lagged panel models can highlight and clarify the complex and dynamic relationship between interpersonal processes and PTSD symptoms. Such analyses demonstrates both bidirectional and unidirectional relations between PSS and PTSD symptoms across time (Woodward et al., 2018). In their study, Freedman and colleagues (2015) included recent civilian trauma survivors (*n* = 501) who sought emergency care. The authors show that changes in social relationship satisfaction in the early phases following exposure to traumatic events, contributed to changes in PTSD, rather than the other way around. They suggest that being satisfied with one’s relationship may be crucial to the natural recovery of trauma as well to be able to benefit from cognitive behavior therapy. Moreover, a postdisaster study among of 658 victims of the Hurricane Ike revealed a significant bidirectional relationship between emotional SS and PTSD symptoms in the early phase from 2–6 months, but no association between the two variables neither 5–9 nor 14–19 months after the incident (Platt et al., 2016). In a study of 557 natural disaster victims from Mexico, Kaniasty and Norris (2008) found that PSS lowered PTSD symptoms in the earlier post disaster phase from 6–12 months (social causation). However, the bidirectional relationship was significant 12–18 months after the event, and at 18 months, were only PTSD symptoms diminished PSS over time (social selection). The relationship was thus altered due to time; PSS closer to the event had greater impact on PTSD symptoms, while the opposite was evident as time went by. Therefore, Kaniasty and Norris (2008) suggest elapsed time to be of importance. Moreover, Ozer and colleagues (2003) concluded that the time interval from exposure to violence affected the strength of the relationship between SS and PTSD symptoms. They argue that SS is a stronger predictor if more than three years have proceeded after the event, compared to less time. Such findings suggest of developmental trajectory to the interaction between SS and PTSD. Wagner and coworkers (2016) found an inverse relationship to PTSD, where the association seemed reinforced across time. In sum, research that analyses the relationship between SS and PTSD symptoms differ in methodology, analytic approaches, and both phenomena are operationalized in several ways. Typically, surveyed populations are a mix of civilians and veterans, and consist of participants exposed to different types of traumatic events (Vogt et al., 2017; Wagner et al., 2016).

In the presented sample of nondomestic victims, we have previously reported a prevalence of 30% (22/73) probable PTSD after one year, and 19% (9/47) after eight years (Johansen et al., 2013). Here, we are interested in adding knowledge to the association between perceived social support and PTSD symptoms across time since only a few longitudinal studies have utilized cross-lagged models testing a three or four assessment points relationship (Shallcross et al., 2016; Woodward et al., 2018).

The main purposes of the present study were therefore to advance the inquiry into the relative importance of social causation or social selection between perceived social support and PTSD symptoms through a period of eight years, and to reflect and discuss clinical implications. We used crossed-lagged models to investigate the relation between PSS and PTSD symptoms in four assessment rounds within the eight-year period after exposure to a physical assault.

## Methods and Materials

### Design

This study is part of a larger prospective investigation utilizing within a one-group design of psychological trajectories in physically assaulted adult victims of a single physical assault, defined as nondomestic violence. Four assessment rounds were performed throughout a period of eight years combined with semistructural interviews at the first round.

**Figure 1.**
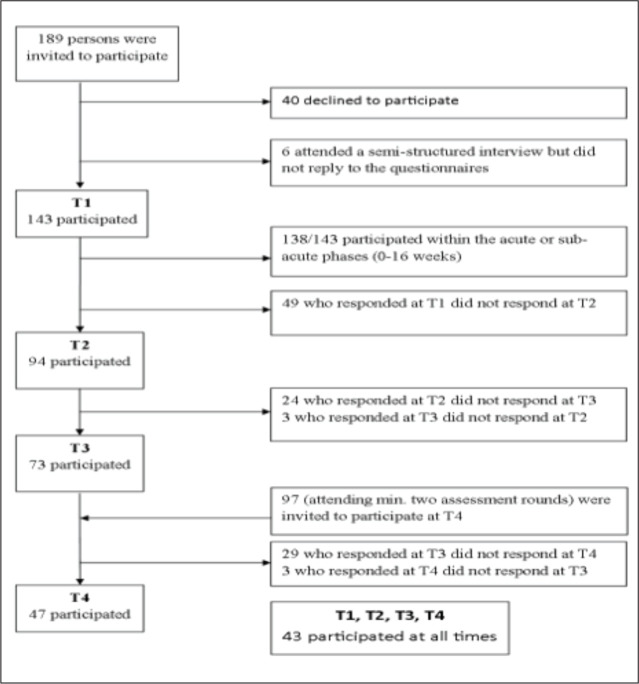
Flowchart for inclusion of participants.

*Participants*. The participants were all victims of physical assault violence; 93% of the perpetrators were strangers and 7% were acquaintances or friends. The victims either sought medical aid, and received health care from a medical emergency unit, or they requested legal aid by submitting a police report at the local police department. With assistance of local police or medical service staff, the participants were recruited from the two largest communities in Norway, the cities of Oslo and Bergen. Of 189 invited victims, 46 declined to participate or did not return the questionnaires, leaving a total sample of 143 (response rate 75.6%). Most participants (138/143) completed the initial questionnaires within 16 weeks after the assault, T1. The second assessment round T2, followed 3 months after T1, the third T3, 12 months after T1, and the last T4, eight years after T1. In total at T1, there were 143 participants, at T2 the response rate was 66% (*n* = 94), at T3 51% (*n* = 73). Written consents were obtained in connection with the conduct of the semistructured interview, either in person or by post. Those who completed at least 2 out of the first 3 assessment rounds (*n* = 97) were reinvited to participate at T4. The response rate at T4 was 48% (*n* = 47), thus representing 33% of the original sample (Figure 1). Fourteen of the 143 invited participants at T3, and 10 of the 97 at T4, could not be reached due to unknown addresses.

Sample characteristics, such as gender, violence category (physical injury), educational level, and level of perceived threat during the assault, are presented for T1 and T4 (Table 1).

**Table 1.** Descriptive Information of Participants at T1 (*n* = 143) and Those Participating at All Assessment Points (*n* = 43).

	Sample at T1 (*n* = 143)		Sample Responding at All Assessment Points (*n* = 43)		Sign. Diff. Dropouts^a^	
	*n*	%	*n*	%	Chi-square	*p* Value
Gender Male Female	114 29	79.7% 20.3%	35 8	81.4 18.6	0.11	.74
Marital status^b^ Married/registered partner Single Divorced/separated	25 101 16	17.6% 71.1% 11.3%	9 29 5	20.9% 67.4% 11.6%	0.51	.77
Education^b^ Elementary school Intermediate-level education Upper-secondary education Higher education, up to 4 years Higher education, more than 4 years	11 50 31 38 12	7.7% 35.2% 21.8% 26.8% 8.5%	2 12 5 18 6	4.7% 27.9% 11.6% 41.9% 14.0%	12.10	.02
Unemployed Yes No	16 127	11.2% 88.8%	3 40	7.0% 93.0%	1.10	.30
Violence category^c^ Assault Inflicting bodily harm	45 98	31.5 68.5%	15 28	34.9 65.1%	0.33	.56
Victim’s perception of threat Felt life was at risk Fear of severe physical injury Understood danger afterward Did not perceive as dangerous	50 25 15 28	42.4% 21.2% 12.7% 23.7%	16 9 4 9	42.1% 23.7% 10.5% 23.7%	0.38	.95
Age (years at T1)	Mean 31	*SD* Range 11.0 18–75				

*Note*. ^a^Significance test of the difference between the sample who participated at all assessment points (*n* = 43) and dropouts at any time point (*n* = 100). ^b^Information is missing for one participant. ^c^The injuries of each participant were classified into these legal categories at T1 in cooperation with the police and in accordance with a judgment based on the level of physical injury and the intention of the perpetrator to cause harm (where physical injury is the most important criterion) (The Norwegian General Civil Penal Code (Straffeloven) Sections 228 and 229) . The assault category includes less serious physical injuries, often combined with threats of more severe physical injury. The victims of inflicted bodily harm include people with more serious physical injuries, ranging from near-fatal injuries to bone fractures or other substantial damage.

At T1, 80% of the participants were males and 20% females, ranging from 18 to 75 years of age (mean = 31 years, *SD* = 11.0). Facial and other head injuries were frequent. Approximately one-third of the sample had serious physical injuries that required specialist treatment beyond the emergency unit. Of those participating in all four assessment rounds, 23% (*n* = 10/43) had received psychiatric treatment afterwards, four by the public health system, four at their workplace, and two by private contacts. Several of the participants reported having sought treatment, without being prioritized within the public health services. Independent sample *t*-tests showed statistically significant differences in mean educational levels between respondents and dropouts (*t* = 2.46, *p* = .01, *df* = 140), where respondents had higher levels of education. No statistically significant differences were found between participants responding at all four assessment rounds (*n* = 43) and dropouts at any time (*n* = 100) in terms of age, gender, level of physical injury, prior experience of violence, cohabitation, marital status, employment, social status, perceived life threat, PTSD symptoms or PSS at T1. For detailed information about the sample, crime characteristics, experiences of prior violence, reported emotions during the assault and acute reactions see (Johansen et al., 2006, 2008). In total, 42% (18/43) who participated in all four-assessment rounds had been exposed to physical violence before recruitment, while 23% (10/43) of them were exposed to new incidents later. For information on exposure to subsequent occurrence of violence and other negative life events during the eight years, see (Johansen et al., 2013, p. 3).

*Ethical approval.* The study was approved by the Regional Committee for Medical and Health Research Ethics, West (REK-West, No. 154.01), and by the Privacy Ombudsman, Norwegian Social Science Data Services (NSD, No. 8750).

### Instruments

*PTSD* *symptoms—Impact* *of Event Scale (IES-22)* is a self-reported questionnaire measuring three core dimensions of stress during the previous 7 days in response to a traumatic event: intrusion (8 items); avoidance (8 items), and arousal (6 items) (Weiss & Marmar, 1997). The items are scored on a 4-point Likert scale; 0 = not at all, 1 = rare, 3 = sometimes, and 5 = often, where a higher score represents higher level of symptom load. The scale, which is related to the psychiatric diagnostic systems ICD-10 (World Health Organization, WHO, 1992) and DSM-IV (American Psychiatric Association, APA, 2000) show excellent psychometric properties (Creamer et al., 2003). The sum score was used in the cross-lagged analysis as previous research has found IES-R (revised), which contains the same items as IES-22, to be essentially unidimensional (Tiemensma et al., 2018). In support, the Omega hierarchical was 0.84 in the present sample for the first assessment round.

*Perceived social* *support—The* *Social Provisions Scale (SPS)* consists of a 24-item questionnaire (Cutrona & Russell, 1987; Perera, 2016) with six provisions (social factors): attachment, social integration, nurturance, reassurance of worth, reliable alliance and guidance. According to Weiss (1974), who developed the provisions; “Attachment,” this is characterized as emotional closeness that attains a sense of security, usually arising from close relationships such as family members or friends. “Social integration” is belonging to a group that share same interests and is often experienced through friendships. “Guidance” consists of advice or information from parents, teachers, or others, while belonging to a family most often gives “Reliable alliance,” which is the need of security of tangible assistance. “Reassurance of self-worth,” described as recognition of competence, skills, and value by others, arise for instance from colleagues at work, while the provision “Opportunity for nurturance” is often described as the sense of others relying on you as one’s offspring or spouse (Cutrona & Russell, 1987; Weiss, 1974). The components of several interpersonal relationships are included in the SPS through these 6 provisions. Each item is scored on a 4-point Likert scale; 1 = strongly disagree, 2 = disagree, 3 = agree, and 4 = strongly agree, where the respondents indicate the extent to which the statements describe their current social relationships. Adding the six subscales together forms a total social provision score representing a general support index (Perera, 2016). Scores are ranged from 1 to 4 for the subscales, and 6 to 24 for the total SPS. The questionnaire has been shown to display high sensitivity and specificity (Perera, 2016). The sum-score for all six subscales was used in the cross-lagged analysis as an index including several social networks and relationships. All six provisions were measured, and the results are presented to describe the contextual span, important for clinical practice.

### Statistical Analysis

**Figure 2.**
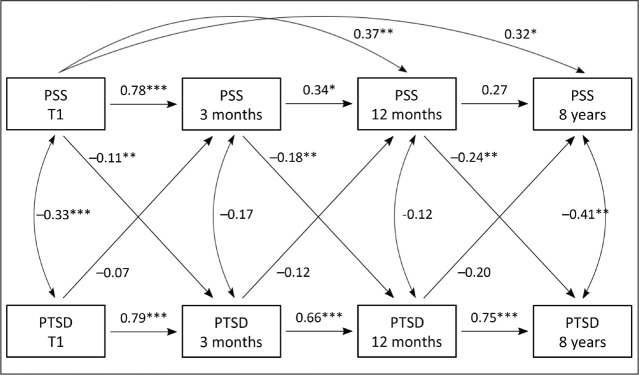
Cross-lagged model showing the relation between PSS and PTSD through four assessment points.

*Note*. *p < .10. **p < .05. ***p < .001. The model shows standardized regression weights through four time points, and covariates at each measure point of PSS and PTSD. PSS = Perceived social support as measured by the Social Provisions Scale (SPS). PTSD = Post-traumatic stress disorder symptoms as measured by the Impact of Event Scale-22 (IES-22). T1= within four months after the assault, T2 = three months after T1, T3 = one year after T1 and T4 = eight years after T1.

Initially, mean values and standard deviation (*SD*) were used to describe the basic features at the four assessment points of the total and the subscale scores of both PTSD symptoms measured by IES-22, and PSS measured by SPS (SPSS package 22). The relationship between PTSD symptoms and PSS across eight years was analyzed using an autoregressive cross-lagged panel model. As shown in Figure 2 (model), the cross-lagged path analyses investigates whether PTSD symptoms are associated with PSS (at a given time point t), after controlling for stability over time (PSS scores at a given time point t (T1, T2, or T3) regressed on the immediately preceding time point). These path analyses also modeled the opposite relationship: whether PSS (at time point t) is associated with PTSD symptoms after controlling for the stability of PTSD symptoms. The cross-lagged panel model was analyzed by the use of the *lavaan* package in R version 3.5.1, including the full information maximum likelihood estimation estimator (FIML) (R Core Team, 2018). Consequently, available observations of participants who had completed the assessments at least on one time-point, were included in the analyses. This is a valid method of handling missing data that are completely missing at random (MCAR), or depends only on the observed data used in the analysis (missing at random; MAR) (Schafer & Graham, 2002). As educational level was related to the probability of dropping out from the study, it was included as an auxiliary variable in the cross-lagged analyses to aid the plausibility of the MAR assumption (Enders, 2008). The overall fit of the models was assessed with: χ^2^ statistics with degrees of freedom and *p* values, root mean square error of approximation (RMSEA) with 90% confidence intervals (CIs) and *p* values, comparative fit index (CFI), and Tucker–Lewis index (TLI) (Browne & Cudeck, 1992; Hu & Bentler, 1999). The cutoff for acceptable model fit has been suggested to be .95 or above for CFI and TLI, and from .06 to .08 or less for RMSEA (Browne & Cudeck, 1992; Hu & Bentler, 1999).

## Results

### Perceived Social Support (PSS) and Post-traumatic Stress Disorder (PTSD) Symptoms

Table 2 shows means and *SD* on total scales and subscales at all assessment points for PSS as measured by the Social Provision Scale (SPS), and PTSD symptoms as measured by the Impact of Event Scale-22 (IES-22). Mean values for the three subscales (symptom clusters) intrusion, avoidance and arousal assessed by IES-22, were found to decline in the same way as total scores over time, but still being much higher than the general population after eight years (for more details see Johansen et al., 2013).

**Table 2.** Descriptive Information on Scales and Subscales at T1, T2, T3, and T4.

	T1		T2		T3		T4	
Scale/*subscales*	Mean	*SD*	Mean	*SD*	Mean	*SD*	Mean	*SD*
IES-22-tot	39.6	27.6	34.9	27.1	32.5	28.9	20.6	25.3
*Intrusion*	14.5	10.6	12.3	10.2	11.2	11.6	6.9	8.9
*Avoidance*	12.3	10.5	9.4	11.8	12.0	11.8	7.2	9.6
*Arousal*	10.4	8.7	9.7	8.8	9.5	8.9	6.5	8.9
SPS-tot	21.4	2.5	21.2	2.2	20.7	3.5	21.7	4.3
*Attachment*	3.6	0.5	3.5	0.6	3.4	0.7	3.6	0.6
*Social integration*	3.6	0.5	3.6	0.5	3.5	0.7	3.6	0.7
*Guidance*	3.6	0.6	3.6	0.6	3.5	0.7	3.5	0.8
*Reassurance of worth*	3.6	0.6	3.6	0.5	3.4	0.7	3.5	0.7
*Opportunity to provide nurturance*	3.2	0.7	3.2	0.7	3.3	0.6	3.3	0.8
*Reliable alliance*	3.7	0.5	3.7	0.5	3.6	0.6	3.7	0.6

*Note*. IES = Post-traumatic stress disorder symptoms as measured by the Impact of Event Scale–22 and perceived social support as measured by Social Provisions Scale (SPS). T1 = within four months after the assault (*n* = 143), T2 = 3 months after T1 (*n* = 94), T3 = 12 months after T1 (*n* = 73), T4 = 8 years after T1 (*n* = 47).

Participants’ interpersonal relationships visualized by the subscores appear to be stable with high values across all assessment rounds. For example, the provision “Attachment” that expresses emotional closeness to for instance family and friends, appears to be stable as well as “Social integration” that represent group affiliation.

Figure 2 (model) and Table 3 depict the analysis of the relationship between PSS and PTSD symptoms through the four assessment points. The cross-lagged model had adequate fit to the data after allowing for PSS at T3 and T4 to be regressed on PSS at T1 (determined by modification indices); robust *χ*^2^ = 10.85; *df* = 10; *p* = .370; robust RMSEA = .027 90% CI = .00–.107; *p* < .001, robust CFI = .998; and robust TLI = .993.

*Stability of PTSD symptoms*. The analysis showed a strong and stable association between the prior scores of PTSD symptoms and later PTSD symptoms through all assessment points. PTSD scores at all-time points were highly correlated (between 0.76 and 0.69, *p* < .001).

*Variability of perceived social support*. Prior PSS scores and later PSS scores between T1↓ T2 and T1↓T3 were highly related (b* = 0.78, *p* < .001 and b* = 0.37, *p* < .05). The relation between T2↓T3 was also statistically significant (b* = 0.34, *p* < .5). The relationship between T1↓T4 was near significant (b* = 0.32, *p* = .054), and T3↓T4 was not significant (b* = 0.27, *p* = .42). The added paths that was included in the modified model revealed that PSS at T1 was statistically significant related to PSS at T3 (b* = 0.37, *p* < .05) and nearly statistically significant related to PSS at T4 (b* = 0.32, *p* < .10) (see Figure 2 [model] and Table 3).

**Table 3.** Regression Weights for the Cross-lagged Model of Perceived Social Support (PSS) and Post-traumatic Stress Disorder Symptoms (PTSD) Over Time.

	Figure 2 Model				
	Standard All	Estimate	Standard Error	*z* Value	*p* Value
PSS T1 → PSS T2	0.78	0.84	0.07	12.7	<.001***
PSS T1 → PSS T3	0.37	0.53	0.24	2.3	.024**
PSS T1 → PSS T4	0.32	0.60	0.31	1.9	.054*
PSS T1 → PTSD T2	−0.11	−0.33	0.16	−2.1	.035**
PTSD T1 → PTSD T2	0.79	0.76	0.07	11.6	<.001***
PTSD T1 → PSS T2	−0.07	−0.02	0.02	−1.3	.188
PSS T2 → PSS T3	0.34	0.46	0.25	1.8	.071*
PSS T2 → PTSD T3	−0.18	−0.51	0.25	−2.05	.041**
PTSD T2 → PTSD T3	0.66	0.72	0.09	7.9	<.001***
PTSD T2 → PSS T3	−0.12	−0.06	0.07	−0.9	.352
PSS T3 → PSS T4	0.27	0.34	0.43	0.8	.424
PSS T3 → PTSD T4	−0.24	−0.47	0.17	−2.7	.006**
PTSD T3 → PTSD T4	0.75	0.69	0.08	8.6	<.001***
PTSD T3 → PSS T4	−0.20	−0.12	0.12	−1.0	.311

*Note*. Standardized regression weights in the cross-lagged model presented in Figure 2. PSS = Perceived social support as measured by the Social Provisions scale (SPS); PTSD = Post-traumatic stress disorder symptoms as measured by the Impact of Event Scale-22 (IES-22); T1 = within 4 months after the assault, T2 = 3 months after T1, T3 = 12 months after T1, T4 = 8 years after T1. *p < .10. **p < .05. ***p < .001.

*Cross-lagged relations.* The relation between prior PSS and later PTSD symptoms were found to be inverse and statistically significant through all assessment points (PSS-T1 ↓ PTSD-T2 b* = –0.33, *p* < .05; PSS-T2 ↓PTSD-T3, b* = –0.51, *p* < .05; PSS-T3 ↓PTSD T4 b* = –0.47, *p* <.05). The relation between prior PTSD symptoms and later level of PSS were also found to be inverse but not statistically significant (PTSD at T1↓PSS-T2 b* = –0.02, *p* = .188; PTSD T2↓PSS-T3 b* = –0.06, *p* = .352; PTSD T3↓PSS-T4 b* = –0.12, *p* = .311).

## Discussion

The present study examined the longitudinal bidirectional relationship between PSS and PTSD symptoms in assault victims across an eight-year period. We find that higher levels of PSS protect against PTSD symptoms, and that lower levels of PSS can increase PTSD symptoms. The opposite, effects of PTSD symptoms on PSS levels, were not significant. However, especially as the relationship is heading in the expected direction, and the relatively small sample size of the study, we cannot rule out the possibility of such an association (Figure 2, model).

The relationship between prior levels of PSS and later levels of PTSD symptoms has a quite similar strength between T1 and T2, as well as for T2 and T3, and even stronger for the third association between one and eight years. Our findings correspond with Ozer and colleagues’ meta-analysis (2003) that revealed stronger associations between SS and PTSD in studies conducted more than three years after the traumatic event, compared to studies with a shorter postassessment period. The inverse influence we found of PSS on PTSD symptoms, across all assessment points supports the causation theories previously introduced (Freedman et al., 2015; Pruitt & Zoellner, 2008). A study of disaster victims additionally give support for the selection model when investigating PSS and PTSD symptoms 12–18 months postexposure, while supporting the causation model when measuring the relationship 6–12 months and 18–24 months postexposure (Kaniasty & Norris, 1993).

We note that in sum, quite a few studies support the selection model (Kaniasty & Norris, 2008). Among U.S. veterans, more severe PTSD symptoms predict later worsening in SS, while levels of SS does not seem to affect future PTSD (Dworkin et al., 2018; King et al., 2006; Laffaye et al., 2008), also shown in torture victims from Iraq (Hall et al., 2014). The inconsistency of findings may be due to differences in the characteristics of the populations studied, i.e., civilian versus military personnel.

We find PSS at T1 to be an independent predictor of PSS at T3 and T4, explained by the rather strong relation between the first PSS assessment close to the exposure to violence, and PSS after one and eight years. Ozer and colleagues’ (2003) suggested a cumulative effect of SS over time, with a growing strength after several years after the exposure, alternatively, that SS function as a secondary prevention. Their explanation is that as PTSD symptoms eventually diminishes and no longer are considered as acute reactions after traumatic exposure, the preventive effect of SS also becomes clearer (Ozer et al., 2003).

In addition, our results of the relationship between PSS at different time points show that the relative stability varied, with a high value between T1 and T2, and low values between T2 and T3 as well as between T3 and T4. This indicate that PSS after 3 months has quite low explanatory value when it comes to PSS one year after the event. Similarly, after one year, PSS has a low explanatory value for PSS after eight years. It is reasonable to suggest that PSS near exposure to violence is more positively expressed from close relationships, such as family and friends, while later PSS has features of more distanced social acquaintances thus affecting the questionnaire scores. Social relations after trauma is a complex issue, and how we seek the benefit of relationships affects the actual availability of social connections, which further influences how we think, feel and act (Bryant, 2016). To understand how post-traumatic stress reactions inflict on a social network context is of importance for illuminating many of the core mechanisms that may influence interpersonal adjustments (Bryant, 2016).

The combination of total PSS scores used in the cross-lagged model, and all six different provisions, give us a broad and inclusive understanding of the participants’ perception of SS. Usually, each provision reflects scores from one type of relationship only, but a person commonly receive several provisions at once (Cutrona & Russell, 1987). The total PSS score were stable with significant relationship through all assessment points as shown in Figure 2 (model), and the mean value of both total scores and subscales showed just small variations as displayed in Table 2. Probably, to get a better understanding of the victim’s need for SS after violence, the context of their involvement in various levels of relationships should be recognized. For example, the sociointerpersonal model introduced by Maerceker and Horn (2013) can be useful to illustrate the importance of PSS for both the specific victim and for the professionals. Several researchers now acknowledge that social network size or density of social contacts does not necessarily equal the actual support provided (Guay et al., 2011; Platt et al., 2014). The level of engagement in social groups and being socially active may have a greater personal impact on mental health than mere perception of the strength of SS. For instance, in their epidemiologic survey (*n* = 31,650), Platt and colleagues (2014) found that a diversity of social networks were protective against PTSD symptoms. Regarding negative or even lack of SS, several studies have suggested that this has a stronger explanatory power than positive support in describing the relationship between PSS and PTSD symptoms (Brewin & Holmes, 2003; Scarpa et al., 2006). Ullman (1996) found negative social reactions to be strongly associated with increased psychiatric symptoms in sexual assaulted victims. The only factor related to better adjustment was being believed in and being listened to by others. More, positive social reactions were unrelated to adjustment (Ullman, 1996). Further, studies among victims of the terror attack at Utøya, Norway, on the 22nd of July 2011, show that SS barriers are highly associated with post-traumatic stress symptoms (Thoresen et al., 2014). Our findings correspond to other longitudinal studies that underline how PSS plays an important role for adult victims by ameliorating and/or protecting against the development of PTSD, however, none of these studies includes a reassessment eight years after the event (Kaniasty & Norris, 2008; Robinaugh et al., 2011; Vogt et al., 2017; Yap & Devilly, 2004).

To feel supported, people seem to need involvement within different levels of social context, and to receive stability through different types of relationships (Maercker & Horn, 2013; Weiss, 1974). Another discussion of relevance is whether PSS is related to the individual personality trait, or to the more dynamic personality state. The first interpretation, recognizes PSS as being consistent and long lasting, thus presupposes stability across time and events. PSS is then a strong personality component imbedded in more traditional attachment theories. Our findings are more consistent with the second comprehension that personality is more dynamic as PSS varies and fluctuates due to recent experiences (Sarason et al., 1990; Yap & Devilly, 2004). Compared to individuals who report low levels of perceived support, those who report high levels of support seem to habit somewhat different coping strategies to overcome their emotional distress (Kaniasty & Norris, 2008).

## Clinical Implications

In clinical contexts, the dynamic characteristics of PSS, as well as the phenomenon of victimization, must be considered. The experience of someone intentionally wanting to inflict injury makes exposure to such violence somewhat different from other types of trauma. Perceived, intention of a harming act is a serious risk factor, which often increases the victim’s negative reactions (Kilpatrick & Acierno, 2003; Sareen, 2014). Exposure to violence as in physical assault, is characterized as interpersonal potential traumatic events (PTEs), and the prospective of preventing long-term mental health problems should be highlighted (Birur et al., 2017; Johansen et al., 2007; Kilpatrick & Acierno, 2003). Assault variables such as the severity of physical injury, characteristics of the assault scenery, and victims level of self-efficacy, may affect the development of PTSD symptoms as well as PSS (Kilpatrick & Acierno, 2003; Nygaard et al., 2017). Females are two to three times more likely to develop PTSD symptoms than males (Olff, 2017), which was also the case from our findings at T1. In total, 86% (24/28) of the female victims and 52% (57/110) of the male victims scored within probable or partial PTSD in the acute phase by IES-15 with values between 35 and 75 for probable, and 20 and 34 for partial (Johansen et al., 2006, Table 2). Andrews et al. (2003) found in a sample of victims of violent crime (118 males and 39 females), that the effect of support satisfaction or negative responses six-month after exposure were significantly enhanced for the females. Despite the well-documented benefits of SS, there are barriers that prevent active use, especially among males. Women seem to adopt SS to a greater extent than men for coping with major life events (Taylor et al., 2000). The use of social networks is an example of such gender difference, where females tend to show more active approach coping strategies compared to males (Larsen, 2011).

The total sample consisting of 80% male participants had a high and stable rate of PTSD symptoms throughout all four assessment points (Table 2). Considering that the sample included several young males (mean age = 31, range = 18–75), being at the peak of their physical and psychological health, should indicate expectations of a high probability of recovery. Instead, the likelihood of developing PTSD symptoms was found to be 48% among those who participated in all rounds (Johansen et al., 2013). The steady and high numbers of PTSD symptoms in a long-term perspective, show the importance of providing sufficient measures to prevent or alleviate the sufferings, regardless of gender. It will be of most interest to gain increased knowledge on how SS can contribute within this context.

Unfortunately, victims of nondomestic violence does not seem to be prioritized or integrated into Norwegian political and public health strategies (Norwegian Ministry of Justice and Public Security, 2014). Not to compete with the necessary attention towards follow-up victims of violence in close relationships, but the many incidents of nondomestic violence in society combined with a significant amount of long-term prevalence of PTSD symptoms, addresses the need to focus upon these victims accordingly.

Our participants consisted of victims who did receive psychiatric treatment or none such treatment after the incident. Of those who completed the assessments at all four rounds, 23% (10/43) had received psychiatric treatment. One might speculate whether high scores on PSS should be considered an important long-term factor in natural recovery after being subjected to a violent assault, as well as representing importance for the outcomes of psychosocial follow-up or psychiatric treatment. Likewise, low PSS might be considered a risk factor among those who did not receive treatment as well as for those who did. A study of social relationship satisfaction associated with PTSD by Freedman et al. (2015), included both treated (98/501) and untreated (313/501) individuals, and the results show similar trend following traumatic events. Hence, it might be appropriate to prioritize interventions aiming at increasing positive SS, as a preventive measure. Further, that clinical practice systematically advise victims of violence and their closed ones to be aware of potentially emerging PTSD symptoms, to reinforce positive social interactions as well as attenuate negative interactions (Guay et al., 2006; Wagner et al., 2016). As held by the causation theories (Freedman et al., 2015; Pruitt & Zoellner, 2008), victims perceptions of available support may be disturbed, both in early stages and in the long run after exposure to PTE. Within a clinical perspective, one should be attentive towards the avoidance effect emerging from PTSD symptoms; avoidance and retraction behavior may drive family and friends away (Andrews et al., 2003). Not to ignore, family and friends may alienate an individual with PTSD, and this should equally be addressed (Ladd & Churchill, 2012).

We believe that proper information to members of formal as well as informal support networks on how both positive and negative SS may influence post-traumatic stress reactions after exposure to physical assault, may increase knowledge and understanding about their ability to respond supportively. This might in turn encourage strategies of recovery making a crucial difference for the affected individual (Ullman & Peter-Hagene, 2016). Within this context, relations and interpersonal communication are typically at stake as time passes. The situation may include distress and uncertainty for the partner, with the potential of secondary traumatization, mistrust, or caregiver burden that might ruin the relationship (Lambert et al., 2012). Regardless of the trauma survivor’s perception of SS being accurate or mistaken, low levels of PSS experiences may render the victim less resistant to the negative psychological impact of significant others after the violence. An informed professional judgment of the victims PSS can be crucial to follow-up decisions (Yap & Devilly, 2004). If a family member displays PTSD symptoms, information about the importance of SS, common PTSD symptoms and potential challenges for all family members, may be beneficial as well as advice to seek help if it becomes particularly demanding. These kind of increased knowledge might prevent or mitigate development of symptoms as well as negative consequences for the family (Dyregrov & Dyregrov, 2008).

Partner accommodation is a relatively new and potentially important construct to consider in treatment planning for people with PTSD (Fredman et al., 2016). When individual perceptions of actual support are mistaken, interventions should include components to change this cognitive scheme, to discourage the negative effects of reduced PSS, for example, by correcting the perception through exposure (Yap & Devilly, 2004). However, family members should be included in follow-up also when perceptions of low support seem correct. It might be warranted to increase support-seeking behavior as well (Kaniasty & Norris, 2008). A better understanding of how PSS affects the course of PTSD in couples’ therapy may be particularly useful (Fredman et al., 2016). Importantly, victims of nondomestic violence do not necessarily seek medical assistance due to physical injuries, and even less psychiatric treatment (McCart et al., 2010). During the acute phase of emotional numbness and cognitive deterioration, it is sometimes difficult to comprehend support from others, and to evaluate need of professional care. One should therefore implement routines at frontline medical units to contact the victims after the initial visit, identifying resources such as social networks and support access. Other potentially harmful post-traumatic reactions such as sleep disturbances, nightmares, changes in eating habits, self-medication, sick leave, or other symptoms related to PTSD should be noted. Involving the partner in clinical practice can be particularly effective for couples where the partner is very aware of PTSD symptoms. Combined with other relevant support measures to relieve such symptoms, it may prevent development of long-term weakening, and improve quality of life and well-being for both the inflicted individual and their significant other.

## Strengths and Limitations

The main strength of our study is the longitudinal design. As far as we are aware, this is the first study being published that includes the assessment of PTSD symptoms and PSS across four different time points after a nondomestic violence event, the last measures conducted eight years later. The study design offers the possibility of investigating the effects of such violence in short, intermediate, and longer terms. By using cross-lagged autoregressive structural equation models, we minimized the undesirable effects of confounders, and were able to address the long-term bidirectional interaction between PTSD symptoms and PSS.

Another strength is the homogeneity of the potential traumatic events; all the respondents were exposed to physical assault by a perpetrator other than a family member. In addition, restrictive inclusion criteria and legally defined definitions of physical injuries were applied, and the gender distribution at T1 was representative to the population experiencing violent crime (other than domestic assault) in Norway (Statistics Norway, 2009; Steen & Hunskaar, 2004). According to the review by Kaniasty & Norris (2008), only a handful of longitudinal studies display similar homogeneity. Naturally, we lack previolence (baseline) measures of PTSD symptoms and PSS. To improve the validity of our study by minimizing recall bias, we strived to perform the interviews at T1 as soon as possible after the event. Furthermore, knowledge of the exact date of exposure and the elapsed time from the exposure to the interview for all participants, strengthens the validity.

One limitation to consider is generalization of the current findings to other groups exposed to serious violence. Our sample, mostly males, were recruited based on the experience of a single physical assault. Further, there is a challenge associated with the complexity of the conceptual and methodological issues within this type of research. However, some individuals are clearly more susceptible to develop PTSD than others (Sareen, 2014; >WHO, 2002). Trauma reactions have some specific symptom characteristics, and at the same time, there are numbers of individual variations (WHO, 2018). Understanding the individual trauma history, personal self-esteem and potential SS will be important in terms of prevention and treatment, regardless of the specific event or gender. Another limitation is the relatively small sample size and a rather high degree of drop out across the four assessments were only 43 of 143 participants completed all four rounds up to the eight-year period. High numbers of participants who drop out is an unfortunate but a common problem in longitudinal studies of assault victims, as motivation to take part diminishes as time passes (Andrews et al., 2003; Elklit & Brink, 2004; Peleg & Shalev, 2006). To include all available observation of all participants at any time and to reduce the drop out effect, we used FIML in the regression model.

We used self-reported questionnaires to measure PTSD symptoms and PSS. Knowing the complexity of these phenomena, the fact that we only measured PSS must be considered a limitation as well. A more thorough understanding of SS and interpersonal interactions requires different types of assessment (Vogt et al., 2017). To compensate for not performing structured clinical interviews, two different questionnaires were used to assess PTSD symptoms at each assessment point, as described in previous articles (Johansen et al., 2013). We found IES-22 to be a valid measure of post-traumatic stress symptoms. Despite these limitations, our findings seem solid. Both questionnaires, IES-22 and SPS, are commonly used for measuring PTSD symptoms and PSS, both are reported to be reliable and valid (Creamer et al., 2003; Cutrona & Russell, 1987; Perera, 2016).

## Conclusion

Regardless of theoretical models, quality and availability of SS seems to play an important role by protecting the victims against developing PTSD after being subjected to a physical assault. Our results support the need to recognize that a single physical assault can have major impacts on the victim’s life for years to come. It is essential to widen this knowledge for the prevention and relief of PTSD symptoms. We also need to identify those at risk for developing serious psychopathology, and to provide relevant public information, victim support, and sufficient treatment.

## Future Research

Interpersonal processes in close relationships, as well as influences from other social networks, represent an important research approach in understanding the interaction between SS and PTSD symptoms (Maercker & Horn, 2013). Future studies should be designed to grasp deeper into the connection between the dimensions of PSS and post-traumatic stress symptoms, and how this connection might coexist for many years or even for life. Furthermore, we need to know more about how to prevent and reduce severe psychopathology for those exposed to nondomestic violence, in the long run.

## Author Biographies

**Venke A. Johansen**, CCN, PhD, is a senior advisor at RVTS West, Haukeland University Hospital and associate professor at the Western Norway University of Applied Sciences, Norway. Her research focuses on among other possible prevention and increased risk of post-traumatic stress reactions after exposure to violence or other potentially traumatic events, as well as consequences for quality of life.

**Anne Marita Milde**, PhD, has a specialist degree in clinical psychology. She is working as a senior researcher at Regional Centre for Child and Youth Mental Health and Child Welfare, NORCE. Her research focuses on among other traumatic stress and psychophysiological stress responses and effects on behavior, with experience from both animal and human studies, thus an interest in identifying mechanisms within a translational perspective.

**Roy Miodini Nilsen**, PhD, is a professor at the Western Norway University of Applied Sciences, Norway. He is a biostatistician with a special focus on longitudinal modeling in epidemiological research. Currently, his research focuses on perinatal health outcomes in immigrant women using large national population-based data.

**Kyrre Breivik**, PhD, is senior researcher at the Regional Centre for Child and Adolescent Mental Health and Welfare, NORCE, the Norwegian Research Centre. His current research interests focus on bullying, child and adolescent development, and children’s adjustment to divorce and measurement issues.

**Dag Øystein Nordanger**, PhD, is professor in psychology at Oslo Metropolitan University and specialist in clinical child psychology at Resource Centre on Violence, Traumatic Stress and Suicide Western Norway (RVTS West). His research and publications have circled around the impacts of trauma in general, with a particular focus the later years on the consequences of childhood adversities and on interventions to prevent and treat them.

**Kjell Morten Stormark**, PhD, is a professor at Regional Centre for Child and Youth Mental Health and Child Welfare, NORCE. His research interests are on psychiatric epidemiology, health promotion and developmental trajectories in children and adolescents.

**Lars Weisæth**, MR, PhD, is emeritus professor of psychiatry (traumatic stress) at the University of Oslo. Since the mid-1970s his research has focused on how humans respond before, during and after life threatening situations such as natural and man-made disasters, war, peacekeeping operations, accidents, terror, violence, and CBRN-incidents.
